# Unusual Conditions Impairing Saliva Secretion: Developmental Anomalies of Salivary Glands

**DOI:** 10.3389/fphys.2019.00855

**Published:** 2019-07-03

**Authors:** Lucrezia Togni, Marco Mascitti, Andrea Santarelli, Maria Contaldo, Antonio Romano, Rosario Serpico, Corrado Rubini

**Affiliations:** ^1^Department of Clinical Specialistic and Dental Sciences, Marche Polytechnic University, Ancona, Italy; ^2^National Institute of Health and Science of Aging, IRCCS INRCA, Ancona, Italy; ^3^Multidisciplinary Department of Medical-Surgical and Dental Specialties, University of Campania “Luigi Vanvitelli”, Naples, Italy; ^4^Department of Biomedical Sciences and Public Health, Marche Polytechnic University, Ancona, Italy

**Keywords:** salivary glands, salivary gland aplasia, accessory salivary gland, accessory salivary ducts, facial developmental anomalies, stem cells

## Abstract

Salivary glands (SG) arise from ectodermal tissue between 6 and 12th weeks of intrauterine life through finely regulated epithelial-mesenchymal interactions. For this reason, different types of structural congenital anomalies, ranging from asymptomatic anatomical variants to alterations associated with syndromic conditions, have been described. Notable glandular parenchyma anomalies are the SG aplasia and the ectopic SG tissue. Major SG aplasia is a developmental anomaly, leading to variable degrees of xerostomia, and oral dryness. Ectopic SG tissue can occur as accessory gland tissue, salivary tissue associated with branchial cleft anomalies, or true heterotopic SG tissue. Among salivary ducts anomalies, congenital atresia is a rare developmental anomaly due to duct canalization failure in oral cavity, lead to salivary retention posterior to the imperforate orifice. Accessory ducts originate from the invagination of the developing duct in two places or from the premature ventral branching of the main duct. Heterotopic ducts may arise from glandular bud positioned in an anomalous site lateral to the stomodeum or from the failure of the intraoral groove development, hindering their proximal canalization. These anomalies require multidisciplinary approach to diagnosis and treatment. While ectopic or accessory SG tissue/ducts often do not require any treatment, patients with SG aplasia could benefit from strategies for restoring SG function. This article attempts to review the literature on SG parenchyma and ducts anomalies in head and neck region providing clinicians with a comprehensive range of clinical phenotypes and possible future applications of bioengineered therapies for next-generation of regenerative medicine.

## Introduction

Salivary glands (SG) arise from ectodermal tissue during intrauterine life following a complex and well-timed sequence of epithelial-mesenchymal interactions (McDonald et al., 1986; Pham Dang et al., 2010). Parotid glands are the first to develop from the 6th week of intrauterine life, followed by submandibular (7th week), sublingual (8th week), and the other minor SGs (9–12th weeks) (Zhang et al., 2010; Berta et al., 2013; Chadi et al., 2017). Whereas parotid and minor SGs are certainly ectodermal in origin, submandibular, and sublingual glands arise from the mouth floor in the junctional regions between ectoderm and endoderm (Amin and Bailey, 2001; Antoniades et al., 2006; Higley et al., 2010). From 18 to 25th week, SG acquire connective capsules and interlobular ducts, while glandular parenchyma shows a rapid growth, leading to highly increase in the number of lobules, and almost complete canalization of the tubules (Aronovich and Edwards, 2014). Although the parotid is the first to develop, it is encapsulated after the submandibular and sublingual glands. This delayed encapsulation is critical because lymphatic system develops within mesoderm after encapsulation of the submandibular and sublingual glands but prior to encapsulation of the parotid gland (Goldenberg et al., 2000). Noteworthy, distal perforation of the submandibular duct (SMD) or Wharton duct into the medial paralingual sulcus is believed to be one of the last stages in the SG development (Amin and Bailey, 2001; Aronovich and Edwards, 2014).

Parotid gland produces almost exclusively serous saliva, while submandibular and sublingual glands produce both serous and mucous secretions (Chadi et al., 2017). Parotid secretions are carried by the main parotid duct (PD) or Stensen duct into oral cavity: it arises from the anterior margin of the gland, from the confluence of two main tributaries, and opens on the parotid papilla into the oral vestibule. The PD mean length and diameter are 50.0 and 1.7 mm, respectively. An accessory PD may join the main duct as the latter passes over the masseter muscle (Kulkarni et al., 2011).

Submandibular duct begins by numerous branches from deep surface of the gland and it drains in a narrow orifice on the top of sublingual caruncle. The SMD mean length and diameter are 62.0 and 3.0 mm, respectively. Along its course, SMD bends at the posterior margin of mylohyoid muscle to form the genu of the SMD (with the angle ranging from 102.7° to 114.4°) (Horsburgh and Massoud, 2013). The major sublingual duct (Bartholin duct) originates from the center of the gland and is accompanied by 8–15 minor excretory ducts (Rivinus ducts). Whereas the minor ducts open into the sublingual fold, Bartholin duct opens alone or in conjunction with SMD at the sublingual caruncle (Zhang et al., 2010).

As previously mentioned, the SG development is a finely regulated process, involving well-timed sequences of cellular, and tissue events. For this reason, different types of structural congenital anomalies have been described so far, ranging from asymptomatic anatomical variants to alterations associated with syndromic conditions (Table 1). The aim of this article is to review the literature on SG parenchyma ad ducts anomalies in head and neck region (Table 2 and Supplementary Table 1), providing clinicians with a comprehensive range of clinical phenotypes, and possible future applications of bioengineered therapies for SG repair and regeneration.

**TABLE 1:** Developmental anomalies and manifestation related to salivary gland aplasia/hypoplasia in syndromic and non-syndromic patients.

**Developmental anomalies**	**Clinical features (head and neck)**
**1. Syndromes closely related to SG aplasia/hypoplasia**	
Lacrimo-auriculo-dento-digital syndrome (LADD) or Levy-Hollister syndrome	Oral cavity: small and sharp lateral incisor, lateral upper incisor agenesis, and bifid uvula
	Lacrimal glands: aplasia/hypoplasia, duct obstruction, and absence of lacrimal punctum
	Other: cup-shaped ears, sensory, or mixed deafness.
Oculo-auriculo-vertebral spectrum (OAVS)	Oral cavity: micrognathia, macrostomia, oral facial cleft, mandibular hypoplasia/deformity, and tongue anomaly;
	Other: microtia, hemifacial macrosomia, facial palsy, branchial cyst, preauricular skin tag, and oral apraxia
Ectrodactyly ectodermal dysplasia cleft lip/palate syndrome (ECC)	Oral cavity: dental abnormalities, lip, and palate cleft Lacrimal glands: absence of lacrimal punctum Other: deformed ears, conductive hearing loss
**2. Syndromes that can be associated with SG aplasia/hypoplasia**	
Down syndrome	Oral cavity: dental agenesia, hypo/hyper/microdontia, delayed eruption, open bite, taurodontism, gingivitis/periodontitis, cheilitis-stomatitis, ogival vault, protruding tongue, and maxillary processes hypoplasia
	Head and neck: slanting palpebral fissures, epicanthic folds, brachycephaly, flat cranial base, and flattened nose bridge
Klinefelter syndrome	Oral cavity: shovel-shaped incisor, taurodontism, and delayed eruption
	Head and neck: brachycephaly
Treacher-Collins syndrome	Oral cavity: mandibular/maxillary dysostosis, cleft palate
**3. Developmental anomalies associated with SG aplasia/hypoplasia in non-syndromic patients**	
Lacrimal glands	Secretion disorders
Oral cavity	Hypo/oligo/anodontia, enamel hypoplasia, multiple caries, fissured tongue, lip, and palate cleft
Head and neck	Cranial deformity, mandibular ramus agenesia

*SG, salivary gland.*

**TABLE 2:** Summary table of developmental disorders involving salivary glands and ducts.

**Developmental disorders**	**Main features**
Major SG aplasia	• Sporadic, familial, or syndromic forms (e.g., LADD, Down syndrome)
	• Isolated or in association with lacrimal gland disorders or other anomalies (e.g., cleft lip and palate)
Parotid gland aplasia	• Sporadic, familial, or syndromic forms (e.g., Klinefelter syndrome)
	• Unilateral or bilateral form
	• Isolated or in association with other gland anomalies (e.g., contralateral parotid gland hypoplasia/hypertrophy, parotid accessory tissue, lacrimal anomalies) or other diseases (e.g., pleomorphic adenoma, angioma)
Submandibular gland aplasia	• Sporadic or syndromic forms (e.g., ectodermal dysplasia)
	• Unilateral or bilateral form
	• Isolated or in association with other gland anomalies (e.g., sublingual gland hypoplasia/hypertrophy, cleft lip, and palate)
Ectopic SG tissue	• Accessory parotid gland (normal anatomical variant) and submandibular gland, with/without accessory salivary ducts
	• Gland tissue associated with branchial cleft anomalies (Huschke foramen)
	• Heterotopic SG tissue, with/without accessory salivary duct and other anomalies, sporadic, or familial form
SD atresia	• Unilateral or bilateral form
	• Atresia of submandibular or sublingual SD
Duplication or accessory SDs	• Unilateral or bilateral form
	• Duplicated or accessory SD (submandibular, sublingual, or parotid gland)
	• Isolated or associated with SG duplication

*SG, salivary glands; SD, salivary ducts; LADD, Lacrimo-auriculo-dento-digital syndrome.*

## Glandular Parenchyma Anomalies

### Aplastic/Hypoplastic Gland Tissue

Aplasia/hypoplasia of the major SGs are rare developmental anomalies, either unilateral or bilateral, that can be isolated or part of a hereditary syndrome (McDonald et al., 1986; Yan et al., 2012). SG aplasia may occur alone or in association with accessory salivary tissue or with other developmental anomalies of the first pharyngeal arch. The first report describing major SGs aplasia was by Bradbury in 1879, while the earliest case of parotid glands aplasia was reported by Poirier in 1881 (Ferguson and Ponnambalam, 2005). The first genetic-related absence of SG was reported in 1924 by Ramsey, who described a pleotropic autosomal dominant disorder, with a high degree of penetrance, more commonly found in males (Matsuda et al., 1999; Goldenberg et al., 2000).

Parotid gland aplasia has an estimated incidence of 1:5000 live births, while only 49 cases of submandibular aplasia have been reported until now. The higher frequency of parotid gland aplasia could be related to its earlier morphogenesis compared to the other SG, exposing this gland to increased risk of developmental impairment (Goldenberg et al., 2000).

Clinical presentation varies depending on the number of missing SGs, their role in salivary flow, and the presence of compensatory hypertrophy of the remaining glands. SG aplasia leads to variable degrees of xerostomia and oral dryness, although some patients with hypoplasia and/or, unilateral aplasia are asymptomatic (McDonald et al., 1986; Matsuda et al., 1999).

Main symptoms include erythematous oral mucosa, glossitis, cheilitis, chronic erythematous candidiasis, and exfoliate lips. There is also an increased risk for dental caries, teeth erosion, periodontal disease, tongue papillary atrophy, pain, and oral ulcers. Furthermore, patients can report hoarseness, dysphagia, oropharyngeal infections, feeding/swallowing impairment, intolerance to acidic or spicy food, dysgeusia, and gastric acid reflux due to inadequate salivary buffering action (Antoniades et al., 2006; Herrera-Calvo et al., 2011; Santarelli et al., 2015).

The intraoral examination reveals the absence of parotid papillae and/or SMD orifices, lack of saliva production upon palpation and, in cases of unilateral aplasia, asymmetry of the parotid/submandibular region. The imaging techniques used to demonstrate SG anomalies include: Ultrasonography, computed tomography (CT), magnetic resonance imaging (MRI), sialography, and scintigraphy (Matsuda et al., 1999). Ultrasonography is a primary step to assess anomalies of superficial parotid lobe and submandibular glands, while MRI is used for deeper lesions or sublingual glands. Sialography is prescribed to assess duct anomalies and ductal system of accessory/rudimentary SGs, while scintigraphy is useful to assess secretory function, including that of the minor SGs of nasal and oropharyngeal regions. Laboratory tests and/or SG biopsy can be obtained in order to determine if the underlying cause is related to systemic diseases, such as Sjögren syndrome (Antoniades et al., 2006; Higley et al., 2010; Herrera-Calvo et al., 2011).

Only topical therapies are available for SG aplasia, based on saliva substitutes. Other measures include lifestyle changes, such as increasing water intake and limiting irritating foods. Furthermore, regular dental examinations, low-sugar diet, and use of fluoride-based dental products are suggested in order to prevent dental caries (Chadi et al., 2017).

Salivary glands aplasia can occur with other multiple facial developmental anomalies (Table 1). The association of parotid and lacrimal aplasia raises the possibility of an ectodermal defect manifesting after the 6th week of fetal life. The absence of submandibular and sublingual glands supports their ectodermal origin although it is impossible to differentiate between ectodermal and endodermal-derived epithelium in the mouth floor (McDonald et al., 1986).

The mutation of FGF-10 or FGFR2b seems to decrease the survival of progenitor cells in a murine model, leading to aplasia/hypoplasia of major SGs (Berta et al., 2013). Although FGF-10 mutation has been reported in some patients, the diagnosis is usually based on clinico-radiological findings. The diagnostic genetic investigations are recommended only if they have a substantial impact on patient management. The mechanisms related to sporadic cases can be either a *de novo* mutation gene, especially FGF-10, or a multifactorial cause associated with genetic and/or environmental factors (e.g., infections during pregnancy) (Berta et al., 2013).

### Ectopic Gland Tissue

The ectopic SG tissue has been reported in several body sites including hypophysis, middle and external ear, mandible, thyroglossal duct, thyroid and parathyroid gland capsules, lymph nodes, and neck region. Ectopic SG tissue can occur in three forms: (a) accessory gland tissue; (b) salivary tissue associated with branchial cleft anomalies; and (c) true heterotopic salivary gland tissue (HSGT).

Finally, stafne bone defect (SBD) should be mentioned as a special case of ectopic SG tissue. This is a bone cavity usually located on the posterior mandible, below the inferior alveolar canal (Philipsen et al., 2002). Several Authors suggested that SBD could be the result of hypertrophic SG lobe, an erosion from vascular tissue compression, or the incomplete Meckel cartilage calcification. Although SBD usually contains ectopic glandular tissue, other tissues can be found in the bone cavity, such as muscles, lymphoid tissue, blood vessels, fat, or connective tissue. Furthermore, even if present, glandular tissue is not directly connected to other SGs nor to independent ductal system (Hisatomi et al., 2019). For these reasons, this anomaly will not be further discussed.

#### Accessory Gland Tissue

Accessory SG tissue refers to lobules of SG tissue which are completely separated from the main gland at a variable distance, but which drain into the main duct. It may arise either from SG inclusions within lymph nodes or from anomalous migration of salivary lobules. The accessory parotid gland is present in about 21% of the population and is considered a normal anatomical variant. The histological features are usually identical to those of the main parotid gland, although 26% of accessory parotid tissue showed both mucous, and serous acini (Antoniades et al., 2006; Higley et al., 2010). The ectopic tissue is more susceptible to SG neoplasms: indeed, 1–7% of all parotid tumors arise from accessory glands, half of which is histologically malignant (Batsakis, 1986; Cannon et al., 2012). On the contrary, accessory submandibular gland is extremely rare; indeed, the first case was reported in 2000 and to date only 4 cases were reported (Koybasioglu et al., 2000).

#### Gland Tissue Associated With Branchial Cleft Anomalies

Branchial cleft anomalies result from persistence of the embryonic branchial cleft apparatus. The Huschke foramen (also known as foramen tympanicum) is a developmental defect of the first branchial cleft affecting 5–46% of the population, consisting in a connection between external acoustic canal and temporomandibular joint (De Zoysa et al., 2009). It has been associated with herniation of soft tissues from the temporomandibular joint into the external auditory meatus. In rare cases, this defect may result in fistula formation between the parotid gland and the external auditory meatus (Rushton and Pemberton, 2005). Since the first case in 1984, only 11 additional cases have been described in literature. Therefore, due to the rarity and non-specific clinical presentation of this anomaly, misdiagnosis is frequent (Rushton and Pemberton, 2005). The diagnosis is made by demonstrating the presence of amylase in the ear discharge, followed by a sialography. CT and MRI are useful to demonstrate bony anomalies and/or fistulous tract through the soft tissue (De Zoysa et al., 2009). Surgical treatment is the only option, consisting in closing the sialo-aural fistula by superficial parotidectomy (Cannon et al., 2012). Although the sialo-aural fistula is not a congenital abnormality *per se*, it is a spontaneous complication of a congenital malformation involving SG tissue.

#### True Heterotopic Salivary Gland Tissue

Heterotopic salivary gland tissue consists in salivary tissue with an independent ductal system located in an unusual anatomical site, isolated from the main SG, and clinically presenting as a saliva-draining skin fistula (Cannon et al., 2012). The external opening can be located in oral mucosa, retroauricular region, buccal, or cervical region (Dutta, 2017). Less extensive surgery is usually required for HSGT because the associated tract, if present, is usually short in contrast to branchial cleft anomalies, which may exist as complete fistulas (Cannon et al., 2012).

The ectopic parotid system refers to a specific anomaly in the parotid gland development, characterized by a congenital saliva-draining cheek fistula near the oral commissure. Congenital salivary fistula results from the partial failure in normal involution of the pharyngeal arches during embryogenesis. The external fistulous orifice typically opens near the mouth angle, at the fusion site of the maxillary and mandibular processes (Gadodia et al., 2008; Dutta, 2017). The constant presence of preauricular appendages and occasional mandibular hypoplasia suggests that this condition could be included in the Oculo-Auriculo-Vertebral spectrum (Dutta, 2017). In 2010 was reported a case of bilateral and symmetrical heterotopic submandibular glands, suggesting that epithelial cord forming the submandibular gland must have bifurcated and persisted to grow as separated SGs (Sanli et al., 2010).

Imaging techniques can be used to demonstrate the existence of accessory parotid gland with a separate ectopic duct, not communicating with the main gland, and duct (Kulkarni et al., 2011). Several surgical procedures have been proposed, mainly the excision of the ectopic accessory parotid with its ductal system or the intraoral transposition of the fistulous tract. Alternatively, less invasive technique consists of Ultrasonography-guided intraglandular injection of botulinum toxin and cauterization of fistula with trichloroacetic acid injection, but an higher risk of infection has been reported (Dutta, 2017).

## Salivary Ducts Anomalies

### Congenital Atresia of Submandibular Duct

Submandibular duct congenital atresia, described for the first time in 1955, is a rare developmental anomaly due to canalization failure of the duct in oral cavity (Mandel and Alfi, 2012). The consequences of this condition are the salivary retention posterior to the imperforate orifice and the presence of cyst-like swelling in the mouth floor (Prosdocimo et al., 2018). Almost all cases observed in newborns, showing male predominance (73%), and unilateral clinical presentation (75%).

Clinical manifestation is a bluish cyst-like swelling containing translucent fluid in the mouth floor following the SMD course. The main differential diagnosis of SMD congenital atresia is oral ranula, and it can be difficult to clinically distinguish between them. However, the histological examination showed lack of epithelial wall, while SMD atresia results in a cystic cavity lined by pseudostratified columnar epithelium with brush borders and thin connective tissue consistent with a dilated duct (Amin and Bailey, 2001; Prosdocimo et al., 2018). The other differential diagnoses include dermal/epidermal inclusion cysts, thyroglossal cysts, bronchogenic cysts, lymphatic and vascular lesions, SG tumors, and sialolithiasis (Pownell et al., 1992; Rosow et al., 2009). MRI is mandatory for differential diagnosis, showing an elongated and dilated tubular structure, segmentally lobed in the mouth floor, consistent with the duct anatomic course. The sphincteric areas appear as localized duct narrowing (Gadodia et al., 2007; Mandel and Alfi, 2012).

Surgical treatment is indicated to avoid the sequelae associated with an imperforate orifice, such as glandular atrophy, permanent duct dilation, sialadenitis, airway compromise and feeding difficulties. The marsupialization is the treatment of choice, while other surgical options include simple incision, excision of terminal portion of the duct with sialodochoplasty, and stenting of the duct (Pownell et al., 1992; Gadodia et al., 2007).

### Duplication and Accessory Salivary Ducts

Cases of PD anomalies are rare. Therefore, there are no adequate reports on its anatomic position and malformations. PD is formed by the confluence of two ducts near the posterior mandibular ramus border. When the two ducts merged with each other outside the parotid gland, the duplication of PD is determined. The histological sections allow to determine the existence of a lumen to confirm the presence of duplication ducts (Aktan et al., 2001; Hassanzadeh Taheri et al., 2015).

Accessory SMD originates from the invagination of the developing duct in two places or from the premature ventral branching of the main duct. It is usually smaller in caliber and runs parallel to the main duct. Only 9 case of SMD duplication were reported and most of them are unilateral and incidentally detected during sialography. As duplication anomalies are mostly asymptomatic, treatment is needed only for disease processes affecting the accessory duct (Gadodia et al., 2007).

### Heterotopia of PD

The parotid bud develops at the ecto-endodermal junction of the developing stomodeum and opens into the oral cavity. If the parotid bud is positioned in an anomalous site lateral to the stomodeum, a cutaneous PD may develop (Grundfast et al., 1987). Another possibility is the formation of a premature communication with the skin, establishing an externally orifice and preventing the development of the parotid papilla into the oral vestibule. A further hypothesis is the failure of the intraoral groove development, hindering the proximal canalization of the duct, or the formation of the parotid papilla (Grundfast et al., 1987).

## Future Perspectives of Stem/Progenitor Cell and Tissue-Based Therapies for SG Repair and/or Regeneration

Several strategies have been proposed to restoring SG functions: (a) autologous SG-derived epithelial stem/progenitor cells isolated from patients and transplanted to replace functionally damaged/lost cells; (b) non-epithelial cells or their bioactive lysates used to trigger the paracrine regenerative effects on remaining glandular cells or to generate new glandular cells; and (c) biomaterials loaded with glandular cells and/or bioactive lysates to mimic *in vivo* SGs (Lombaert et al., 2017).

The first study for transplanting autologous glandular cells was carried out in rodents using c-Kit+ (CD117) epithelial cells. This research showed that SGs contain cells with stem/progenitor properties that could maintain themselves and differentiate into multiple glandular cell types. Several c-Kit+ cell subpopulations (CD24+, CD49f+, and SCA1+) are located within the major ducts and possess higher levels of stem/progenitor activity. Furthermore, c-Kit+ cells are present in human SGs and can be isolated and cultured *ex vivo*; suggesting future applications for cell therapy. Salivary acinar cells could also be considered for cell therapy as they are able to duplicate themselves to repair local damage (Nanduri et al., 2013).

Salivary glands repair can also be driven by the remaining endogenous stem/progenitor cells. For this reason, any type of transplanted epithelial cell could enhance local endogenous repair if the appropriate molecular stimuli are presented to dormant stem/progenitor cells. Recent efforts to increase the number of c-Kit+ cells *ex vivo* using growth factors may be useful, although the number of cells needed to trigger SG regeneration remains unclear (Pringle et al., 2016).

Recently, the transplantation of a bioengineered germ, with or without mesenchyme, was proposed as a promising strategy to replace SGs in a mouse model with SG defects (Tanaka et al., 2018). Authors demonstrated that SGs orthotopical transplanting led to successful glandular development *in vivo*, showing connection of the new SG to the main duct, and correct formation of ductal and acinar structures. Morphological, immunohistochemical, and genetic analyses confirmed the development of fully functional SG tissue. The orthotopically engrafted SGs were also functional in saliva secretion through the reconstruction of neural network. Another interesting finding was the non-essential role played by mesenchyme in inducing maturation of transplanted SG (Tanaka et al., 2018).

In conclusion, SG anomalies are a heterogeneous group of rare anomalies that requires a multidisciplinary approach to diagnosis and treatment. While ectopic or accessory SG tissue/ducts often do not require any treatment, patients with SG aplasia/hypoplasia could benefit from strategies for restoring SG function. From this perspective, restoring physiological functions with bioengineered organs is expected to be the next-generation of regenerative medicine.

## Author Contributions

LT, MM, AS, and AR conceived the literature review of the manuscript. MM, LT, and MC described the glandular parenchyma anomalies. RS, MC, and CR described the salivary duct anomalies. AS, RS, CR, and AR wrote the concluding remarks of the manuscript. All authors discussed and approved the final version of the manuscript.

## Conflict of Interest Statement

The authors declare that the research was conducted in the absence of any commercial or financial relationships that could be construed as a potential conflict of interest. The handling Editor is currently organizing a Research Topic with one of the authors, AS, and confirms the absence of any other collaboration.
