# Supplementary material for: Unusual Conditions Impairing Saliva Secretion: Developmental Anomalies of Salivary Glands
Source: Front Physiol. 2019 Jul 3;10:855. doi: 10.3389/fphys.2019.00855 (PMC6617833; doi:10.3389/fphys.2019.00855)
Supplement: Supplementary file 1 [file Table_1.docx]

**Supplementary Table 1.** Literature review of salivary gland parenchyma and ducts anomalies. SG = Salivary gland; PG = Parotid gland; SMG = Submandibular gland; SLG = Sublingual gland; PD = Parotid gland; SMD = Submandibular gland. Complete reference list is at the end of the table.

| **Author** | **Year** | **Main features** |
| --- | --- | --- |
| 1. ***Major SGs anomalies*** | | |
| Bradbury EP | 1879 | Major SGs aplasia |
| Ramsey WR | 1924 | Major SGs and lacrimal puncta aplasia (familial form) |
| Blackmar BB | 1925 | Major SGs aplasia |
| Rule DC | 1969 | Major SGs aplasia |
| Smith NJD, Smith PB | 1977 | Major SGs aplasia (familial form) |
| McDonald FG et al. | 1986 | Major SGs aplasia and lacrimal glands hypoplasia |
| Higashino H et al. | 1987 | Major SGs and lacrimal puncta aplasia |
| Whyte AM, Hayward MWJ | 1989 | Major SGs aplasia |
| Higasa R et al. | 1989 | Major SGs aplasia |
| Tokumime H et al. | 1990 | Major SGs aplasia |
| Milunsky JM et al. | 1990 | Major SGs aplasia, lacrimal glands hypoplasia and SMG hypofunction |
| Ashikagaya M et al. | 1991 | Major SGs aplasia |
| Myers MA et al. | 1994 | Major SGs aplasia and impaired lacrimal secretion |
| Gelbier MJ, Winter GB | 1995 | Major SGs and lacrimal puncta aplasia |
| Matsuda C et al. | 1999 | Major SGs aplasia and cleft lip and palate |
| Ferreira PS, Gomez RS | 2000 | Major SGs and lacrimal puncta aplasia (familial form) |
| Ferguson MM, Ponnambalam Y | 2005 | Major SGs aplasia (Down syndrome) |
| Kwon SY et al. | 2006 | Major SGs and lacrimal puncta aplasia (familial form) |
| Heath N et al. | 2006 | Major SGs aplasia |
| Champan DB et al. | 2009 | Major SGs and lacrimal glands aplasia (LADD syndrome) |
| Gok F et al. | 2010 | Major SGs and lacrimal glands aplasia |
| Pham Dang et al. | 2010 | Major SGs aplasia |
| Taji SS et al. | 2011 | Major SGs aplasia |
| Yan Z et al. | 2012 | Major SGs aplasia and unilateral lacrimal puncta aplasia |
| Mohan RP et al. | 2013 | Major SGs aplasia |
| Berta E. et al. | 2013 | Major SGs aplasia (LADD syndrome) |
| Odeh MM et al. | 2013 | Major SGs aplasia (Down syndrome) |
| Chadi MJ et al. | 2017 | Major SGs aplasia (Down syndrome) |
| 1. ***PG aplasia*** | | |
| Poirier H | 1881 | Bilateral PG aplasia |
| Raison J | 1925 | Bilateral PG aplasia |
| Schreiber H | 1927 | Unilateral PG aplasia and contralateral PG hypoplasia |
| Nayak UV, Ayyar AA | 1927 | Bilateral PG aplasia |
| de Morales-Grey J | 1937 | Unilateral PG aplasia |
| Kabakov BD | 1949 | Unilateral PG aplasia |
| Vogel VC | 1978 | Bilateral PG and SMG aplasia and lacrimal canaliculi atresia |
| Wiesenfeld D et al. | 1983 | Bilateral PG aplasia (familial form) |
| Wiesenfeld D et al. | 1985 | Unilateral PG aplasia (familial form) |
| Kelly SA et al. | 1990 | Unilateral PG aplasia and contralateral PG sialosis |
| Almadori G et al. | 1997 | Unilateral PG aplasia and controlateral PG hypertrophy |
| Bhide VN, Warshawsk RJ | 1998 | Unilateral PG aplasia and ispilateral accessory parotid tissue |
| Sichel JY et al. | 1998 | Unilateral PG aplasia and first branchial cleft cyst |
| Gomez RS et al. | 1998 | Bilateral PG and lacrimal puncta aplasia |
| Hyang SJ et al. | 1999 | Unilateral PG aplasia and contralateral PG hypertrophy and ductal atresia |
| Martinez Subias J et al. | 2000 | Unilateral PG aplasia and contralateral PG hypertrophy |
| Goldenberg D et al. | 2000 | Bilateral PG aplasia and bilateral accessory parotid tissue |
| Daniel SJ et al. | 2003 | Unilateral PG aplasia and contralateral PG hypertrophy |
| Martin-Granizo R, Garcia-Gonzales D | 2004 | Unilateral PG aplasia and contralateral PG hypertrophy |
| Salvinelli F et al. | 2004 | Unilateral PG aplasia, ispilateral angioma and thyroid hypoplasia |
| Karakoc O et al. | 2005 | Unilateral PG aplasia and contralateral PG pleomorphic adenoma |
| Kim SH et al. | 2005 | Bilateral PG, SMG and lacrimal glands aplasia with occluded lacrimal puncta |
| D’Ascanio L et al. | 2006 | Unilateral PG aplasia and ispilateral cheek angioma and thyroid hypoplasia |
| Antoniades DZ et al. | 2006 | Bilateral PG aplasia and accessory parotid tissue |
| Al-Talabani N et al. | 2008 | Bilateral PG aplasia |
| Lee BH et al. | 2010 | Unilateral PG aplasia and ispilateral pleomorphic adenoma |
| Higley MJ et al. | 2010 | Bilateral PG aplasia and accessory parotid tissue |
| Yilmaz YF et al. | 2010 | Bilateral PG aplasia (Klinefelter syndrome) |
| Chen Y-K et al. | 2011 | Unilateral PG aplasia |
| Udall S, Cho SY | 2011 | Unilateral PG aplasia |
| Capaccio P et al. | 2012 | Unilateral PG aplasia and accessory parotid tissue |
| Seith AB et al. | 2013 | Unilateral PG aplasia and pleomorphic adenoma of ipsilateral accessory parotid tissue |
| Sun L et al. | 2013 | Unilateral PG aplasia, facial cleft and accessory parotid gland |
| Gunbey HP et al. | 2014 | Unilateral PG aplasia and contralateral PG hypertrophy |
| Ozcelik D et al. | 2014 | Unilateral PG aplasia and ipsilateral facial cleft, accessory mandible and facial weakness |
| Teymoortash A, Hoch S | 2016 | Unilateral PG aplasia and contralateral cheek lipoma |
| 1. ***SMG aplasia*** | | |
| Grϋber W | 1885 | Bilateral SMG aplasia |
| Bruno et al. | 1894 | Unilateral SMG aplasia |
| Nayak UV, Ayyar AA | 1927 | Unilateral SMG aplasia |
| Abdel-Dayem HM | 1978 | Unilateral SMG aplasia |
| Kubo S et al. | 1990 | Unilateral SMG aplasia |
| Abe SK et al. | 1990 | Unilateral SMG aplasia |
| Garcia-Consuegra L et al | 1999 | Unilateral SMG aplasia |
| Yilmaz MD et al. | 2002 | Unilateral SMG aplasia |
| Fracaro MS et al. | 2002 | Bilateral SMG aplasia |
| Singh P et al. | 2004 | Bilateral SMG aplasia (ectodermal dysplasia) |
| Roh JL | 2006 | Unilateral SMG aplasia |
| Koo et al. | 2006 | Unilateral SMG aplasia |
| Srinivasan A et al. | 2006 | Unilateral SMG aplasia and ipsilateral SLG hypertrophy |
| Shipchandler TZ et al. | 2008 | Unilateral SMG aplasia |
| Mathison CC, Hudgins PA | 2008 | Bilateral SMG aplasia and SLG hypertrophy |
| Gallego L et al. | 2009 | Unilateral SMG aplasia |
| Ahmed M et al. | 2009 | Bilateral SMG aplasia and SLG hypertrophy |
| Herrera-Calvo G et al. | 2011 | Unilateral SMG aplasia and ispilateral SLG hypertrophy |
| Hahtanir A | 2012 | Bilateral SMG aplasia and bilateral SLG hypertrophy |
| Garcia Reija et al. | 2013 | Bilateral SMG aplasia, SLG hypertrophy and cleft lip and palate |
| Bhoil R et al. | 2016 | Unilateral SMG aplasia |
| 1. ***Other SG anomalies*** | | |
| Youngs LA, Scofield HH | 1967 | Heterotopic SG tissue in the lower neck |
| Singer MI et al. | 1979 | Heterotopic SG tissue in the neck |
| Goodman RS et al. | 1981 | Heterotopic SG tissue and branchial cleft sinus |
| Sharma PD and Dawkins RS | 1984 | Huschke foramen |
| Yamasaki H et al. | 1986 | Heterotopic PG with cheek fistula |
| Janke PG, Rivon RP | 1988 | Huschke foramen |
| Janke PG, Rivron PR | 1988 | Huschke foramen |
| Hawke M et al. | 1988 | Huschke foramen |
| Heffez L et al. | 1989 | Huschke foramen |
| Rodgers GK et al. | 1990 | Heterotopic SG tissue in the neck, with pleomorphic adenoma |
| Panda NK et al. | 1990 | Huschke foramen |
| Wang RG et al. | 1991 | Huschke foramen |
| Zhao K et al. | 1992 | Heterotopic PG with cheek fistula and ispilateral preauricular appendage |
| Moon WK et al. | 1995 | Heterotopic PG with cheek fistula and ispilateral preauricular appendage |
| Lassaletta-Atienza L et al. | 1998 | Heterotopic SG tissue in the lower neck |
| Köybaşioğlu A et al. | 2000 | Accessory SMG with WD obstruction |
| White DK et al. | 2001 | Accessory salivary tissue |
| Gerhards F et al. | 2001 | Heterotopic SG tissue |
| Kawahara K et al. | 2001 | SMD dilatation |
| Lee DH | 2002 | Heterotopic PG with buccal mucosa fistula |
| Chilla R | 2002 | Huschke foramen |
| Tasar M, Yetiser S | 2003 | Huschke foramen |
| Langer J, Begall K | 2004 | Huschke foramen |
| Rushton VE and Pemberton MN | 2005 | Huschke foramen |
| Hsu RF et al. | 2006 | Heterotopic SG tissue with remnants of II branchial cleft (familial form) |
| Gadodia A et al. | 2007 | Accessory SMG and SMD |
| Gadodia A et al. | 2008 | Accessory PG with congenital cheek fistula and ispilateral preauricular appendage |
| Hah JK et al. | 2008 | Heterotopic PG with cheek fistula and ispilateral preauricular appendage |
| De Zoysa N et al. | 2009 | Huschke foramen |
| Sanli EC et al. | 2010 | Bilateral heterotopic SMG in the upper neck |
| Pinto FR et al. | 2012 | Heterotopic PG with cheek fistula and ispilateral preauricular appendage |
| Cannon DE et al. | 2012 | Heterotopic SG tissue in the lower neck |
| Bryan S et al. | 2013 | Accessory SMG and pleomorphic adenoma |
| Natasha S | 2014 | Heterotopic PG with intraoral opening |
| Desai RS et al. | 2015 | Accessory SMG and pleomorphic adenoma |
| Sun ZP et al. | 2015 | Heterotopic PG with cheek fistula, ispilateral preauricular appendage and mandibular hypoplasia |
| Dutta M | 2016 | Heterotopic PG with cheek fistula |
| Nayak SB | 2018 | Accessory SMG |
| 1. ***Salivary duct anomalies*** | | |
| Rose BH | 1932 | SMD duplication |
| Scher LB, Scher I | 1955 | Bilateral SMD orifice atresia |
| Beke et al. | 1963 | SLD orifice atresia |
| Myenson M et al. | 1966 | Bilateral SMD duplication |
| Dore P, Dozin A | 1968 | Unilateral SMD orifice atresia |
| Forethic EA | 1973 | Bilateral SMD orifices atresia |
| Jafeck BW, Strifo JL | 1973 | SMG lobe duplication |
| Rahmothulla M | 1973 | Accessory SMD |
| Hoggings GS, Hutton JB | 1974 | SLD orifice atresia |
| Towers JF et al. | 1977 | SMD duplication |
| Addante RR | 1984 | Unilateral SMD orifice atresia |
| Mori S et al. | 1986 | Accessory SMD |
| Grundfast KM et al. | 1987 | Cutaneous PD orifice and ispilateral preauricular skin tags |
| Pownell PH et al. | 1992 | Unilateral SMD orifice atresia |
| Pownell PH et al. | 1992 | Bilateral SMD orifice atresia |
| Pownell PH et al. | 1992 | Unilateral SMD duplication |
| Codjambopoulo P et al. | 1992 | SMD and SMG duplication |
| Chen SR et al. | 2000 | SMD duplication |
| Amin MA, Bailey BMW | 2001 | SMD orifice atresia |
| Hoffrichter MS et al. | 2001 | Bilateral SMD orifice atresia |
| Aktan ZA et al. | 2001 | Unilateral PD duplication |
| Walker P | 2005 | SMD orifice atresia |
| Capaccio P et al. | 2007 | Bilateral SMD atresia |
| Pal K, Abdulla AM | 2007 | Congenital SMD orifice atresia |
| Ulualp SO et al. | 2007 | Bilateral SMD orifice atresia |
| Gadodia A et al. | 2007 | Accessory SMD and SMG |
| Samantha PP et al. | 2007 | Ectopic accessory PD |
| Rosow DE et al. | 2009 | Unilateral SMD atresia |
| Fernandens ACS et al. | 2009 | PD duplication |
| Ellegard E, Kjellmer I | 2010 | SMD orifice atresia |
| Astik RB, Dave UH | 2011 | Bilateral PD duplication |
| Mandel L, Alfi D | 2012 | Unilateral SMD orifice atresia |
| Aronovich S, Edwards SP | 2014 | Bilateral SMD orifice atresia |
| Taheri MHD et al. | 2015 | Unilateral PD duplication |
| Prosdocimo ML et al. | 2018 | SMD orifice atresia |

**References.**

Abdel-Dayem, H.M. (1978). Congenital absence of submaxillary gland detected on 99mTc-pertechnetate thyroid imaging. *Clin Nucl Med* 3, 442.

Abe, S.K., Ureshino, T., and Oka, M. (1990). Aplasia of the submandibular gland. A case report. *J Craniomaxillofac Surg* 18, 119-21.

Addante, R.R. (1984). Congenital cystic dilation of the submandibular duct. *Oral Surg* 59, 656-8.

Ahmed, M., Strauss, M., Kassaie, A., Shotelersuk, V., and DeGuzman, R. (2009). Bilateral submandibular gland aplasia with clinico-radiological mass due to prolapsing sublingual salivary tissue through mylohyoid boutonniere: A case report and review. *Dentomaxillofac Radiol* 38, 121-4.

Aktan, Z.A., Bilge, O., Atamaz Pinar, Y., and Omer Ikiz. A. (2001). Duplication of the parotid duct a previously unreported anomaly. *Surg Radiol Anat* 23, 353-4

Almadori, G., Ottaviani, F., Del Ninno, M., Cadoni, G., De Rossi, G., and Paludetti, G. (1997). Monolateral aplasia of the parotid gland. *Annals of Otology, Rhinology & Laryngology* 106, 522-5.

Al-Talabani, N., Gataa, I.S. and Latteef, S.A. (2008). Bilateral agenesis of parotid salivary glands, an extremely rare condition: report of a case and review of literature. *Oral Surgery, Oral Medicine, Oral Pathology, Oral Radiology and Endodontology* 105, e73-5.

Amin, M.A. and Bailey, B.M.W. (2001). Congenital atresia of the orifice of the submandibular duct: a report of 2 cases and review. *British Journal of Oral and Maxillofacial Surgery* 39, 480-2.

Antoniades, D.Z., Markopoulos, A.K., Deligianni, E., and Andreadis, D. (2006). Bilateral aplasia of parotid glands correlated with accessory parotid tissue. *Journal of Laryngology and Otology* 120, 327-9.

Aronovich, S. and Edwards, S.P. (2014). A case of imperforate Wharton duct. *J. Oral Maxillofac. Surg* 72, 744-7.

Ashikagaya, M., Hagiwara, K., Hayashi, T., et al. (1991). Congenital absence of the major salivary glands: report of a case. *Dental Radiology* 31, 143.

Astik, R.B. and Dave, U.H. (2011) Embryological basis of bilateral double parotid ducts: a rare anatomic variation. *Int J Anat Vari* 4, 141-3.

Beke A.L., Tomaro A.J., and Stein M. (1963). Congenital atresia of sublingual duct with ranula: report of case, *J Oral Surg Anesth Hosp Dent Serv* 21 427-8.

Berta, E., Bettega, G., Jouk, P.S., Billy, G., Nugues, F., and Morand, B. (2013). Complete agenesis of major salivary glands. *Int J Pediatr Otorhinolaryngol* 77, 1782-5.

Bhide, V.N. and Warshawsky, R.J. (1998). Agenesis of the parotid gland: association with ipsilateral accessory parotid tissue. *American Journal of Roentgenology* 170, 1670-1.

Bhoila, R., Mistrya, K.A., Bhoilb, R., and Thakurc, K. (2016). Unilateral submandibular depression. *European Annals of Otorhinolaryngology, Head and Neck diseases* 133, 285-7.

Blackmar, B.B. (1925). Congenital atresia of all lacrimal puncta with absence of salivary glands. *Am J Ophthalmol* 8, 139-40.

Bradbury, E.P. (1879). Absence of saliva. *Boston Med Surg J* 100, 342.

Bruno, P. (1894). Mancanza assoluta della ghiandola sottomascellare destra nell’uomo. *Atti del’XI Congresso Medico* 2, 62-3.

Bryan, S., Bodner, L., Manor, E., and Brennan, P.A. (2013). Pleomorphic adenoma occurring outside the submandibular gland: a case report of an accessory submandibular gland. *J Oral Maxillofac Surg 71,* 1703-5.

Cannon, D.E., Szabo, S., and Flanary, V.A. (2012). Heterotopic salivary tissue. *American Journal of Otolaryngology–Head and Neck Medicine and Surgery* 33, 493-6.

Capaccio, P., Gaini, L.M., and Pagani, D. (2008). Videosialendoscopic assessment of bilateral atresia of Wharton’s duct orifice in an infant. *J Pediatr Surg* 42, E5.

Capaccio, P., Luca, N., Sigismund, P.E., and Pignataro, L. (2012). Recurrent inflammation of accessory parotid tissue associated with unilateral parotid gland aplasia: diagnostic and therapeutic implications. *European Archives of Oto-Rhino-Laryngology* 269, 1551-4.

Chadi, M.J., Saint Georges, G., Albert, F., Mainville, G., Nguyen, J.M., and Kauzman, A. (2017). Major salivary gland aplasia and hypoplasia in Down syndrome: review of the literature and report of a case”. *Clinical Case Reports* 5, 939-44.

Chapman, D.B., Shashi, V., and Kirse, D.J. (2009). Case report: aplasia of the lacrimal and major salivary glands (LADD). *Int J Pediatr Otorhinolaryngol* 73, 899-901.

Chen, S.R., Li, W.B., and Zhu, W. (2000). The double ducts of the submandibular gland-1 Case. *Chin J Clin Anat* 18, 88.

Chen, Y.K., Kuo, C.J., and Yeh, CL. (2011). Unilateral agenesis of the parotid gland with contralateral compensation hypermetabolism of FDG. *Clinical Nuclear Medicine* 36, 710-1.

Chilla, R. (2002). Otosialorrhoe. *HNO* 50, 943-5.

Codjambopoulo, P., Ender-Griepekoven, I., and Broy, H. (1992). Bilateral duplication of the submandibular gland and the submandibular duct. *Rofo* 157, 185-6.

D’Ascanio, L., Cavuto, C., Martinelli, M., and Salvinelli, F. (2006). Radiological evaluation of major salivary glands agenesis. A case report, *Minerva Stomatologica*, 55, 223-8.

Daniel, S.J., Blaser, S., and Forte, V. (2003). Unilateral agenesis of the parotid gland: an unusual entity. *International Journal of Pediatric Otorhinolaryngology* 67, 395-7.

de Moraes-Grey, J. (1937). Un cas d’aplasie de la parotide gauche. A propos d’une contribution à l’étude de la région parotidienne [A case of aplasia of the left parotid gland based on a study of the parotid region]. *Ann Anat Pathol* 14, 871-3.

De Zoysa, N., Vasani, S., Kaniyur, S., and Frosh, A. (2008). Gustatory otorrhoea: a rare case of congenital external ear salivary fistula. *The Journal of Laryngology & Otology*, E11.

Desai, R.S., Meshram, D., Jangam, S.S. and Singh, J.S. (2015). Pleomorphic adenoma of an accessory submandibular salivary gland: a rare entity. *Br J Oral Maxillofac Surg*. 53, e33-5.

Dore, P. and Dozin, A. (1968). False ranula of the newborn or imperforation of Wharton’s duct. *Rev Stomatol Chir Maxillofac* 69, 219-26.

Dutta, M. (2017). The Ectopic Accessory Parotid System With Congenital Cheek Fistula: An Overview and Current Update. *Laryngoscope* 127, 1351-60.

Ellegard, E. and Kjellmer, I. (2010). Atresia of the submandibular duct orifices: An unusual cause of feeding problems and failure to thrive in an infant. *Acta Paediatr* 7, 1100.

Ferguson, M.M., and Ponnambalam, Y. (2005). Aplasia of the parotid gland in Down syndrome. *Br J Oral Maxillofac Surg* 43, 113-7.

Fernandes, A.C.S., Lima, R.G., Rossi, M.A., and Aguiar, M.C. (2009). Parotid gland with double duct: an anatomic variation description. *Int J Morphol* 27, 129-32.

Ferreira, A.P., Gomez, R.S., Castro, W.H., Calixto, N.S., Silva, R.A., and Aguiar, M.J. (2000). Congenital absence of lacrimal puncta and salivary glands: report of a Brazilian family and review. *Am J Med Genet* 94, 32-4.

Foretich, E.A. (1973). Bilateral congenital absence of the submandibular duct orifices*. J Oral Surg* 31, 556-7.

Fracaro, M.S., Linnett, V.M., Hallett, K.B., and Savage, N.W. (2002). Submandibular gland aplasia and progressive dental caries: A case report. *Aust Dent J* 47, 347-50.

Gadodia, A., Seith, A., Neyaz, A., Sharma, R., and Thakkar, A. (2007). Magnetic resonance identification of an accessory submandibular duct and gland: an unusual variant. *The Journal of Laryngology & Otology* E18.

Gallego, L., Junquera, L., Cuesta, P., and Rosado, P. (2009). Symptomatic unilateral submandibular gland aplasia. *Br J Oral Maxillofac Surg* 47, 243.

Garcıa Reija, M.F., Patricia Lopez Gordillo, D.P., Blasco Palacio, J.C., Bermejo Abascal, L., and Garcıa-Montesinos Perea, B. (2013). Bilateral Submandibular Gland Aplasia With Hypertrophy of the Sublingual Glands of a Patient With a Cleft Lip and Palate: Case Report. *The Journal of Craniofacial Surgery* 24, 5.

García-Consuegra, L., Gutiérrez, L.J., Castro, J.M., and Granado, J.F. (1999). Congenital unilateral absence of the submandibular gland. *J Oral Maxillofac Surg* 57, 344-6.

Gelbier, M.J., and Winter, G.B. (1995). Absence of salivary glands in children with rampant dental caries: report of seven cases. *Int J Paediatr Dent* 5, 253-7.

Gerhards, F., Büttner, R., and Janicke, S. (2001) Aberrant salivary gland tissue as a diVerential diagnosis of branchiogenic cysts: case report. *HNO* 49, 476-8.

Gok, F., Mutlu, F.M., Sari, E., Demirkaya, E., Altinsoy, H.I., and Bernd, W. (2010). Congenital absence of salivary and lacrimal glands accompanied by growth and development retardation. *J Pediatr Ophthalmol Strabismus* 47, e1-3.

Goldenberg, D., Flax-Goldenberg, R., Joachims, H.Z., and Peled, N. (2000). Misplaced parotid glands: bilateral agenesis of parotid glands associated with bilateral accessory parotid tissue. *J Laryngol Otol* 114, 883-5.

Gomez, R.S., Aguiar, M.J., Ferreira, A.P., and Castro, W.H. (1998). Congenital absence of parotid glands and lacrimal puncta. *J Clin Pediatr Dent* 22, 247-8.

Goodman, R.S., Daly, J.F., and Valensi, Q. (1981). Heterotopic salivary tissue and branchial cleft sinus. *Laryngoscope* 91, 260-4.

Gruber, W. (1885). Congenitaler Mangel beider Glandulae submaxillares bei einem wohlgebildeten, erwachsenen Subjecte. A*rchiv f¨ur Pathologische Anatomie und Physiologie und f¨ur Klinische Medicin* 102, 9-11.

Grundfast, K.M., Barber, C.S., and Kubicki, S.P. (1987). Congenital aberrantly located cutaneous Stensen’s duct orifice-a newly described anomaly. *International Journal of Pediatric Otorhinolaryngology* 14, 197-202.

Gunbey, H.P., Gunbey, E., Tayfun, F., and Kaytez, S.K. (2014). A rare cause of unilateral parotid gland swelling: compensatory hypertrophy due to the aplasia of the contralateral parotid gland. *Journal of Craniofacial Surgery* 25, e265-7.

Hah, J.H., Kim, B.J., Sung, M.W., and Kim, K.H. (2008). Chemocauterization of congenital fistula from the accessory parotid gland. *Clin Exp Otorhinolaryngol* 1, 113-5.

Haktanır, A. (2012). CT and MR findings of bilateral submandibular gland aplasia associated with hypertrophied symmetrical sublingual glands herniated through mylohyoid defects. *Dentomaxillofacial Radiology* 41, 79-83.

Hassanzadeh Taheri, M.M., Afshar, M., and Zardast, M. (2015). Unilateral duplication of the parotid duct, its embryological basis and clinical significance: a rare cadaveric case report. *Anat Sci Int* 90, 197-200.

Hawke, M., Kwok, P., Shankar, L., and Wang, R.G. (1988). Spontaneous temporomandibular joint fistula into the external auditory canal. *J Otolaryngol* 17, 29-31.

Heath, N., Macleod, I., and Pearse, R. (2006). Major salivary gland agenesis in a young child: consequences for oral health. *Int J Pediatr Dent* 16, 431-4.

Heffez, L., Anderson, D., and Mafee, M. (1989). Developmental defects of the tympanic plate: Case reports and review of the literature. *J Oral Maxillofac Surg* 47, 1336-40.

Herrera-Calvo, G., García-Montesinos-Perea, B., Saiz-Bustillo, R., Gallo-Terán, J., and Lastra-García-Barón, P. (2011). Unilateral submandibular gland aplasia with ipsilateral sublingual gland hypertrophy presenting as a neck mass. *Med Oral Patol Oral Cir Bucal* 16, e537-40.

Higasa, R., Uchimura, N., Shinshi, H., et al. (1989). Congenital aplasia of the major salivary glands: a case report. *The Japanese Journal of Pediatric Dentistry* 27, 764.

Higashino, H., Tsugumo, H., Ohkusa, Y., Ohkuma, H., Ino, C., Nakazawa, M., Izumi, H., and Kobayashi, Y. (1987). Congenital absence of lacrimal puncta and of all major salivary glands: case report and literature review. *Clin Pediatr* 26, 366-8.

Higley, M.J., Walkiewicz, T.W., Miller, J.H., Curran, J.G. and Towbin, R.B. (2010). Aplasia of the parotid glands with accessory parotid tissue. *Pediatric Radiology* 40, 345-7.

Hoffrichter, M.S., Obeid, G. and Soliday, J.T. (2001). Bilateral submandibular duct atresia: Case report. *J Oral Maxillofac Surg* 59, 445.

Hoggins, G.S. and Hutton, J.B. (1974). Congenital sublingual cystic swellings due to imperforate salivary ducts. Two case reports. *Oral Surg. Oral Med. Oral Pathol* 37, 370-3.

Hsu, R.F., Hsu, Y.C. and Huang, S.C. (2006) Hereditary ectopic salivary gland: survey of three generations. *Acta Otolaryngol* 126, 330-3.

Hyang, S.J., Gyo, J.K., Yu, C.K. and Soo, K.K. (1999). Unilateral parotid glandular aplasia and ductal atresia. *Korean Journal of Otorhinolaryngology* 42, 377-9.

Jafek, B.W. and Strife, J.L. (1973). Accessory lobe of the submandibular gland. *Radiology* 109, 75-7.

Janke, P.G., and Rivron, R.P. (1988). An unusual case of otorrhoea due to parotid salivary fistula. *Br J Radiol* 61, 509-11.

Kabakov, B.D. (1949). A case of congenital absence of the parotid and defective development of the remaining salivary glands. *Vjestnik Chirurgiyi (Grekov)* 69, 42-3.

Karakoc, O., Akcam, T., Kocaoglu, M., and Yetiser, S. (2005). Agenesis of the unilateral parotid gland associated with pleomorphic adenoma of the contralateral parotid gland. *Journal of Laryngology and Otology* 119, 409-11.

Kawahara, K., Hotta, F., and Miyachi, H. (2000). Congenital dilation of the submandibular duct: Report of a case. *J Oral Maxillofac Surg* 58, 1170.

Kelly, S.A., Black, M.J., and Soames, J.V. (1990). Unilateral enlargement of the parotid gland in a patient with sialosis and contralateral parotid aplasia. *Br J Oral Maxillofac Surg* 28,409-12.

Kim, S.H., Hwang, S., Kweon, S., Kim, T.K. and Oh, J. (2005). Two cases of lacrimal gland agenesis in the same family - clinicoradiologic findings and management. *Can J Ophthalmol* 40, 502-5.

Koo, B.S., Lee, S.W., Lee, Y.M. and Koh, Y.W. (2009). Sialolithiasis in a stump of Wharton’s duct of an aplastic unilateral submandibular gland. *Int J Oral Maxillofac Surg* 38, 91-7.

Köybaşioğlu, A., Ileri, F., Gençay, S., Poyraz, A., Uslu, S. and Inal, E. (2000). Submandibular accessory salivary gland causing Warthin's duct obstruction. *Head Neck* 22, 717-21.

Kubo, S., Abe, K., Ureshino, T. and Oka, M. (1990). Aplasia of the submandibular gland. A case report. *J Craniomaxillofac Surg* 18, 119-21.

Kwon, S.Y., Jung, E.J., Kim, S.H., and Kim, T.K. (2006). A case of major salivary gland agenesis. *Acta Otolaryngol* 126, 219-22.

Langer, J. and Begall, K. (2004). Otosialorrho – Diagnostik und Therapie einer Speichel-fistel des außeren Gehorganges. *Laryngo-Rhino-Otol* 83, 606-9.

Lassaletta-Atienza, L., López-Ríos, F., Martín, G., Benito, A., Bronchalo, F., Martínez-Tello, F.J., and Alvarez-Vicent, J.J. (1998). Salivary gland heterotopia in the lower neck: a report of five cases. *Int J Pediatr Otorhinolaryngol* 43, 153-61.

Lee, B.H. (2010). Unilateral agenesis of the parotid gland associated with a pleomorphic adenoma in the ipsilateral buccal space. *Japanese Journal of Radiology* 28, 224-6.

Lee, D,H. (2002). A case of buccal abscess; originating from an ectopic accessory parotid gland*? J Laryngol Otol* 116, 312-3.

Mandel, L. and Alfi, D. (2012). Diagnostic Imaging for Submandibular Duct Atresia: Literature Review and Case Report. *American Association of Oral and Maxillofacial Surgeons J Oral Maxillofac Surg* 70, 2819-22.

Martınez Subıas, J., Royo Lopez, J., and Valles Varela, H. (2000). Congenital absence of major salivary glands. *Acta Otorrinolaringologica Espanola* 51, 276-8.

Martin-Granizo, R. and Garcia-Gonzalez, D. (2004). Unilateral agenesis of parotid gland: a case report. *Oral Surg Oral Med Oral Path Oral Radiol Endod* 98, 712-4.

Mathison, C.C. and Hudgins, P.A. (2008). Bilateral submandibular gland aplasia with hypertrophy of sublingual glands. *Otolaryngol Head Neck Surg* 138, 119-20.

Matsuda C, Matsui Y, Ohno K, and Michi K. (1999). Salivary gland aplasia with cleft lip and palate. A case report and review of the literature. *Oral Surg Oral Med Oral Pathol Oral Radiol Endod* 87, 594-9.

McDonald, F.G., Mantas, J., McEwen, C.G., and Ferguson, M.M. (1986). Salivary gland aplasia: an ectodermal disorder? *J Oral Pathol* 15, 115-7.

Milunsky JM1, Lee VW, Siegel BS, and Milunsky A. (1990). Agenesis or hypoplasia of major salivary and lacrimal glands. *Am J Med Genet* 37, 371-4.

Mohan, R.P., Verma, S., Chawa, V.R., and Tyagi, K. (2013). Non-syndromic non-familial agenesis of major salivary glands: a report of two cases with review of literature. *J Clin Imaging Sci* 3, 2.

Moon, W.K., Han, M.H., and Kim, I.O. (1995). Congenital fistula from ectopic accessory parotid gland: diagnosis with CT sialography and CT fistulography. *AJNR Am J Neuroradiol* 16, 997-9.

Mori, S., Wada, T., and Harada, Y. (1986). Accessory duct in the submandibular gland. *Oral Surg Oral Med Oral Pathol* 62, 607.

Myenson, M., Crelin, E.S., and Smith, H.W. (1966). Bilateral duplication of the submandibular ducts. *Arch Otolaryngol* 83, 488.

Myers, M.A., Youngberg, R.A., and Bauman, J.M. (1994). Congenital absence of the major salivary glands and impaired lacrimal secretion in a child: case report. *J Am Dent Assoc* 125, 210-2.

Natasha, S. (2014). Congenital parotid fistula. *J Indian Soc Pedod Prev Dent* 32, 357-61.

Nayak, S.B. (2018). Accessory Submandibular Salivary Gland Forming a "Horseshoe" With the Main Submandibular Salivary Gland: A Unique Variation. *J Craniofac Surg* 29, 1376-7.

Nayak, U.V. and Ayyar, A.A. (1927). Anomalies in the development of salivary glands. *J Anat* 61, 261-2.

Odeh, M., Hershkovits, M., Bornstein, J., Loberant, N., Blumenthal, M., and Ophir, E. (2013). Congenital absence of salivary glands in Down syndrome. *Arch Dis Child* 98, 781-3.

Ozcelik, D., Toplu, G., Turkseven, A., Senses, D.A., and Yigit, B. (2014). Lateral facial cleft associated with accessory mandible having teeth, absent parotid gland and peripheral facial weakness. *Journal of Cranio-Maxillofacial Surgery* 42, e239-44.

P. Walker. (2005). Imperforate submandibular duct, *Otolaryngol Head Neck Surg*. 132, 653-4.

Pal, K. and Abdulla, A.M. (2007). Congenital imperforate submandibular duct in a newborn, *Indian J Pediatr* 74, 687-8.

Panda, N.K., Verma, A., Mann, S.B.S., and Mehra, Y.N. (1990). Indeterminate salivary fistula through the external auditory canal. *Ear Nose Throat J* 69, 657-9.

Pham Dang, N., Picard, M., Mondie, J. M., and Barthelemy, I. (2010). Complete congenital agenesis of all major salivary glands: a case report and review of the literature. *Oral Surg Oral Med Oral Pathol Oral Radiol Endod* 110, e23-7.

Pinto, F.R. (2012). A case of congenital fistula from an accessory parotid gland: diagnosis and treatment. *Ear Nose Throat J* 91, 34-6.

Poirier, H. (1881). Absence des parotides [Absence of the parotids]. *Bull Soc Anat (Paris)*, 198-9.

Pownell, P.H., Brown, O.E., and Pransky, S.M. (1992). Manning SC. Congenital abnormalities of the submandibular duct. *Int J Pediatr Otorhinolaryngol* 24, 161-9.

Prosdócimoa, M.L., Barreto Nogueiraa, A.P., de Albuquerque Cavalcantea, M.A., Agostinib, M., de Andradeb, B.A.B., and Romañachb, M.J. (2018). Congenital dilatation of the submandibular duct*. International Journal of Pediatric Otorhinolaryngology* 113, 16-8.

Rahmothulla, M, (1973). A rare case of accessory duct in submandibular sialography. *J Indian Dent Assoc* 45, 563.

Raison, J. (1925). Absence congenitale des parotides. Malformation d’origine heredo-syphilitique. *Rev Stomatol* 27, 340-2.

Ramsey, W.R. (1924). A case of hereditary congenital absence of the salivaryglands. *Am J Dis Child* 28, 440.

Rodgers, G.K., Felder, H., and Yunis, E.J. (1990). Pleomorphic adenoma of cervical heterotopic salivary gland tissue: case report and review of neoplasms arising in cervical heterotopic salivary gland tissue. *Otolaryngol Head Neck Surg* 104, 533-6.

Roh, J.L. (2006). Unilateral submandibular gland aplasia: an isolated phenomenon of early fetal development. *Otolaryngol Head Neck Surg* 135, 332-4.

Rose, B.H. (1932). Bifunction of the submaxillary duct. *Am J Surg* 17, 257.

Rosow, D.E., Ward, R.F., and April, M.M. (2009). Sialodochotomy as treatment for imperforate submandibular duct: A systematic literature review and report of two cases. *Int J Pediatr Otorhinolaryngol* 12, 1613.

Rule, D.C. (1969). Agenesis of salivary glands. *Israel Journal of Dental Medicine* 18, 31.

Rushton, V.E. and Pemberton, M.N. (2005). Salivary otorrhoea: a case report and a review of the literature. *Dentomaxillofacial Radiology* 34, 376-9.

Salvinelli, F., Marte, C., and D’Ascanio, L. (2004). Congenital aplasia of the parotid gland with omolateral cheek angioma: case report and review of the literature. *Acta Oto-Laryngologica* 124, 328-30.

Samanta, P.P., Rana, K.K., Khan, R.Q., and Das, S. (2007). An unusually located human accessory parotid gland. A case report. *Braz J Morphol Sci* 24, 53-4.

Sanli, E.C., Öztürk, N.C., Polat, A., and Öztürk, H. (2010). Bilateral and symmetrical heterotopic submandibular glands in the upper neck: case report. *Surg Radiol Anat* 32, 979-82.

Scher, L.B. and Scher, I. (1955). Case of imperforate submandibular ducts in an infant. *Br Dent J* 98, 324-5.

Schreiber, H. (1927). Rechtsseitige Parotisaplasie bei linksseitiger Parotis dystrophie [Right-sided paratid aplasia and left-sided parotid dystrophy]. *Anat Anz* 63, 349-53.

Seith, A.B., Gadodia, A., Sharma, R., and Parshad, R. (2013). Unilateral parotid agenesis associated with pleomorphic adenoma of ipsilateral accessory parotid gland. *Ear, Nose and Throat Journal* 92, E13-5.

Sharma, P.D. and Dawkins, R.S. (1984). Patent foramen of Huschke and spontaneous salivary fistula. *Laryngol Otol* 98, 83-5.

Shipchandler, T.Z. and Lorenz, R.R.R.R. (2008). Unilateral submandibular gland aplasia masquerading as cancer nodal metastasis. *Am J Otolaryngol* 29, 432-4.

Sichel, J.Y., Halperin, D., Dano, D., and Dangoor, E. (1998). Clinical update on type II first branchial cleft cysts. *Laryngoscope* 108, 1524-7.

Singer, M.I., Applebaum, E.L., and Loy, K.D. (1979). Heterotopic salivary tissue in the neck. Laryngoscope 89, 1772-8.

Singh, P. and Warnakulasuriya, S. (2004). Aplasia of submandibular salivary glands associated with ectodermal dysplasia. *J Oral Pathol Med* 33, 634-6.

Smith, N.J.D. and Smith, P.B. (1977). Congenital absence of major salivary glands. *Br Dent J* 142, 259-60.

Srinivasan, A., Moyer, J.S., and Mukherji, S.K. (2006). Unilateral submandibular gland aplasia associated with ipsilateral sublingual gland hypertrophy. *AJNR Am J Neuroradiol* 27, 2214-6.

Sun, L., Sun, Z., and Ma, X. (2013). Partial duplication of the mandible, parotid aplasia and facial cleft: a rare developmental disorder. *Oral Surgery, Oral Medicine, Oral Pathology and Oral Radiology* 116, e202-9.

Sun, Z.P., Hong, X., Ma, X.C., Zhang, Z.Y., and Yu, G.Y. (2015). Cheek fistula from the ectopic salivary gland: a variant of the oculo-auriculo-vertebral spectrum. *Laryngoscope* 125, 360-4.

Taji, S.S., Savage, N., Holcombe, T., Khan, F., and Seow, W.K. (2011). Congenital aplasia of the major salivary glands: literature review and case report. *Pediatr Dent* 33, 113-8.

Tasar, M. and Yetiser, S. (2003). Congenital salivary fistula in the external auditory canal associated with chronic sialoadenitis and parotid cyst. *J Oral Maxillofac Surg* 61, 1101-4.

Teymoortash, A. and Hoch, S. (2016). Congenital Unilateral Agenesis of the Parotid Gland: A Case Report and Review of the Literature. *Hindawi Publishing Corporation. Case Reports in Dentistry Volume*, Article ID 2672496, 5 pages.

Tokumine, H., Yoshida, S., Araki, M., and Iguchi, T. (1990). Congenital aplasia of salivary glands: report of a case. *Journal of the Japanese Stomatological Society* 39, 800-1.

Towers, J.F. (1977). Duplication of the submandibular salivary duct. *Oral Surg Oral Med Oral Pathol* 44, 326.

Udall, D. and Cho, S.Y. (2011). Congenital agenesis of right parotid gland confounds MIBG scan interpretation in craniocervical neuroblastoma. *Clinical Nuclear Medicine* 36, e162-4.

Ulualp, S.O., Rodriguez. S.C., and Hernandez, J. (2007). Bilateral atresia of the submandibular duct orifices. *Am J Otolaryngol* 28, 184.

Vogel, V.C. and Reinchart, P. (1978). Aplasie der Glandulae Parotides und submandibularis mit Atresie der Canaliculi lacrimales. *Deutsche Zahnärztliche Zeitschrift* 33, 415-7.

Wang, R.G., Bingham, B. Hawke, M., Kwok, P., and Li, J.R. (1991). Persistence of the foramen of Huschke in the adult: an osteological study. *J Otolaryngol* 20, 251-3.

White, D.K., Davidson, H.C., Harnsberger, H.R., Haller, J., and Kamya, A. (2001). Accessory salivary tissue in the mylohyoid boutonnière: a clinical and radiologic pseudolesion of the oral cavity. *AJNR Am J Neuroradiol* 22, 406-12.

Whyte, A.M., and Hayward, M.W.J. (1989). Agenesis of the salivary glands: a report of two cases. *Br J Radiol* 62, 1023-6.

Wiesenfeld, D., Ferguson, M.M., Allan, C.J., McMillan, N.C., and Scully, C. (1983). Bilateral parotid gland aplasia. *Br J Oral Surg* 21, 175-8.

Wiesenfeld, D., Ferguson, M.M., Hardman, F.G., Iverson, E.S., McMillan, N.C., and Sagar, J.A. (1985). Familial parotid gland aplasia. *J Oral Med* 40, 84-5.

Yamasaki, H., Tashiro, H., and Watanabe, T. (1986). Congenital parotid gland fistula. *Int J Oral Maxillofac Surg*. 15, 492-4.

Yan, Z., Ding, N., Liu, X., and Hua, H. (2012). Congenital agenesis of all major salivary glands and absence of unilateral lacrimal puncta: A case report and review of the literature. *Acta Oto-Laryngologica* 132, 671-5.

YIlmaz, M., Yucel, A., Derekoy, S., and Altuntas, A. (2002). Unilateral aplasia of the submandibular gland. *European Archives of Otorhino-Laryngology* 259, 554-6.

Yilmaz, Y.F., Titiz, A., Yurur-Kutlay, N., Ozcan,, M., and Unal, A. (2010). Congenital bilateral parotid gland agenesis in Klinefelter syndrome. *Journal of Cranio-Maxillofacial Surgery* 38, 248-50.

Youngs, L.A. and Scofield, H.H. (1967). Heterotopic salivary gland tissue in the lower neck. *Arch Path* 83, 550-6.

Zhao, K., Wang, L.M., and Qi, D.Y. (1992). Congenital extraoral fistula from an auxiliary parotid gland. *J Oral Maxillofac Surg* 50, 752-3.
